# General transcription factor IIIAa regulates the transcription of somatic 5S rRNA and influences embryonic organ development in zebrafish

**DOI:** 10.1007/s42995-025-00334-5

**Published:** 2025-12-15

**Authors:** Junzhi Luo, Binbin Tao, Ji Chen, Yanlong Song, Wei Hu

**Affiliations:** 1 Key Laboratory of Freshwater Animal Breeding, Ministry of Agriculture and College of Fisheries, Huazhong Agricultural University, Wuhan, 430070 China; 2 State Key Laboratory of Breeding Biotechnology and Sustainable Aquaculture, Hubei Hongshan Laboratory, National Aquatic Biological Resource Center, Institute of Hydrobiology, Chinese Academy of Sciences, Wuhan, 430072 China

**Keywords:** Zebrafish, *gtf3aa*, 5S rRNA, Ribosome, Organ development

## Abstract

Transcription factor IIIA (TFIIIA) is a zinc finger protein that facilitates the assembly of a transcription complex by recruiting transcription factors TFIIIB and TFIIIC, along with RNA polymerase III, to initiate the transcription of 5S rRNA genes. However, the effects of TFIIIA knockout in vertebrates remain unclear. To address this, we investigated the function of a homologous general transcription factor IIIAa, *gtf3aa*, identified as a maternal factor in zebrafish. During early embryonic development, *gtf3aa* expression initially increased and subsequently declined. At 12 h postfertilization, *gtf3aa* mRNA was detected at notably low levels in the embryo, whereas by three days postfertilization, its mRNA level gradually increased in the larvae. The *gtf3aa* was broadly expressed in various embryonic tissues of zebrafish, with higher expression levels observed in the brain, heart, liver, and muscle. Knockout of *gtf3aa* significantly suppressed somatic 5S rRNA transcription in early zebrafish embryos and larvae, resulting in a reduction in the number of mature monoribosomes and polyribosomes. The *gtf3aa*^*−/−*^ larvae exhibited slow growth and delayed yolk absorption, along with impaired development of the eyes, heart, swim bladder, liver, and intestinal tissues. Additionally, expression of genes involved in metabolic signaling pathways, including the peroxisome proliferator-activated receptor pathway, was reduced. The *gtf3aa*^*−/−*^ zebrafish did not survive beyond seven days postfertilization. This study offers preliminary insights into the role of *gtf3aa* in regulating somatic 5S rRNA transcription and embryonic organ development in zebrafish.

**Supplementary Information:**

The online version contains supplementary material available at 10.1007/s42995-025-00334-5.

## Introduction

Transcription factor IIIA (TFIIIA) is a member of the C2H2-type zinc finger family. TFIIIA binds to the regulatory region of 5S ribosomal RNA (5S rRNA) and recruits TFIIIB, TFIIIC, and RNA polymerase III to assemble a transcription complex that initiates 5S rRNA transcription (Talyzina et al. 2023). In addition, TFIIIA directly binds to 5S rRNA to form 7S ribonucleoprotein particles (7S RNP), which are stored in oocytes as a source of material for ribosome assembly (Romaniuk 2005). Most investigations into the regulatory role of TFIIIA in 5S rRNA transcription have been performed in vitro, with only limited studies conducted in vivo. In humans, TFIIIA has been shown to induce expression of the RIG-I ligand RNA5SP141, which activates the IFN-I-mediated immune response and enables the host to defend against herpes simplex virus infection (Naesens et al. 2022). Moreover, Gtf3a promotes colorectal cancer progression by upregulating cystatin A expression through direct binding to the promoter of the cystatin A gene (*Csta*) (Wang et al. 2022). However, the effects of Gtf3a knockout in animals remain poorly defined.

In vertebrates, two distinct types of tandemly arranged 5S rRNA genes have been identified: somatic 5S rRNA, expressed in somatic cells and testes, and maternal 5S rRNA, expressed in oocytes (Allison et al. 1995; Haig 2021; Szymański et al. 2003). The 5S rRNA is a structural component of the large ribosomal subunit and is essential for ribosomal assembly and function. Proper ribosomal assembly is critical for normal tissue development. Previous studies have shown that mutations in various ribosome biogenesis genes can lead to impaired liver development and function (Danilova et al. 2011; Zhao et al. 2014; Zhu et al. 2021). Conditional knockout of the key ribosome biogenesis *Def* in mouse hepatocytes results in abnormal liver cell morphology and persistent infiltration of inflammatory cells (Huang et al. 2020). Similarly, *def* mutations in zebrafish (*Danio rerio*) lead to defective liver development (Chen et al. 2005). The *utp4* gene encodes a protein that is an essential component of the ribonucleoprotein complex responsible for rRNA processing and small subunit assembly. Mutations in zebrafish *utp4* have been shown to cause liver cirrhosis (Wilkins et al. 2013). Defects in the *sbds* result in reduced numbers of 80S ribosomes in zebrafish, which exhibit stunted growth and atrophy of the pancreas, liver, and intestine (Oyarbide et al. 2020). Mutations in the ribosome biogenesis *bms1* impair the development of the liver, exocrine pancreas, and intestines in zebrafish (Wang et al. 2012). Mutations in genes such as *mycn*, *urb1*, and *ltv1*, which are involved in ribosome synthesis, have been associated with developmental defects and delayed formation of digestive organs, including the intestines (He et al. 2017; Li et al. 2022; Zhang et al. 2021). These studies highlight the essential roles of ribosome biogenesis genes in vertebrate tissue development.

In this study, we analyzed the sequence features of Gtf3aa, the protein encoded by general transcription factor IIIAa (*gtf3aa*) in zebrafish, and characterized its spatiotemporal expression pattern and regulatory function in 5S rRNA transcription. A *gtf3aa* knockout mutant was generated, which revealed that the loss of *gtf3aa* significantly disrupted 5S rRNA transcription and ribosome biogenesis in zebrafish larvae. Additionally, *gtf3aa* deficiency impaired yolk absorption and hindered the development of the liver and intestine, ultimately leading to lethality.

## Materials and methods

### Zebrafish maintenance

Zebrafish (AB strain) were housed and bred following the standardized conditions and procedures described in the *Zebrafish Book* (Westerfield 2000). All experimental procedures involving zebrafish were conducted in compliance with the *Guidelines for the Care and Use of Experimental Animals*, as approved by the Institute of Hydrobiology, Chinese Academy of Sciences.

### Sequence alignment and phylogenetic analysis

Protein sequences of Gtf3a homologs from 29 species, including mammals, birds, reptiles, amphibians, and fish, were retrieved from the NCBI (https://www.ncbi.nlm.nih.gov) and ENSEMBL (https://www.ensembl.org) databases (Supplementary Table S1). Amino acid sequences from all species were aligned using the MUSCLE algorithm in MEGA11 (Tamura et al. 2021). A phylogenetic tree was generated using the maximum likelihood method based on the JTT matrix-based model (Jones et al. 1992), with bootstrap analysis performed over 1000 replicates. The phylogenetic tree was visualized using iTOL (Letunic and Bork 2024). Additionally, homologous alignments of Gtf3a amino acid sequences from eight species, including zebrafish, were conducted using ClustalW (Madeira et al. 2024), and the alignment results were visualized with ESPript 3.0 (Robert and Gouet 2014).

### Generation of gtf3aa mutant zebrafish

*Gtf3aa* knockout zebrafish were generated using the CRISPR/Cas9 system. The gRNA targeting *gtf3aa* was designed using the online tool available at https://chopchop.cbu.uib.no. Primer sequences used for gRNA template amplification and mutation detection are listed in Supplementary Table S2. gRNA was synthesized using an in vitro transcription kit (AM1344; Invitrogen, Carlsbad, CA, USA). Both the gRNA and Cas9 protein (A36497; Invitrogen) were microinjected into one-cell stage zebrafish embryos to create F0 mutants. These F0 mutants were subsequently crossed with wild-type fish to generate the F1 generation. Wild-type (WT) and homozygous F2 zebrafish were obtained through intercrossing F1 *gtf3aa*^+*/−*^ fish.

### Larval morphology and histological analysis

The morphology and growth of zebrafish larvae were recorded using a stereomicroscope (Stemi508; Zeiss, Oberkochen, Germany) and analyzed with ImageJ software (https://imagej.net/software/imagej/). Histological examination of liver and intestinal tissues was performed using paraffin-embedded sections stained with hematoxylin and eosin.

### Phenotypic rescue experiment

The full-length *gtf3aa* coding sequence (CDS) was amplified and cloned into the pCS2 vector (primers were listed in Supplementary Table S2). *gtf3aa* mRNA was synthesized using the SP6 Transcription Kit (AM1340; Invitrogen) and purified with the RNeasy Mini Kit (74,104; QIAGEN, Germany). A solution containing 200 ng/µL of *gtf3aa* mRNA and 0.1% phenol red was injected into one-cell stage embryos obtained from *gtf3aa*^+/−^ intercrosses. Embryos injected with vehicle (solvent and 0.1% phenol red) served as controls. Phenotypic analysis of larvae was conducted three days postinjection.

### Quantitative real-time PCR (qPCR)

Total RNA was extracted from zebrafish eggs, embryos at various stages [0.1, 1, 3, 6, 9, 12, 24 h postfertilization (hpf)], whole larvae [2, 3, 4, 5 days postfertilization (dpf)], and tissues from 5 dpf larvae (brain, eyes, heart, intestine, liver, and muscle) using TRIzol Reagent (15,596,026; Invitrogen). RNA of acceptable quality was reverse transcribed into complementary DNA (cDNA) using a commercial reverse transcription kit (R323; Vazyme, Nanjing, China). Gene expression levels were quantified using SYBR qPCR Master Mix (Q712; Vazyme) in accordance with the manufacturer’s protocols. Expression was calculated via the 2^(− ∆Ct) method (Livak and Schmittgen 2001), using 18S rRNA and *β-actin* as internal controls. qPCR and reverse transcription primers for zebrafish 5S and 18S rRNA were obtained from Locati et al. (2017), and all primers used are listed in Supplementary Table S3.

### Oil Red O staining

Neutral lipid staining in zebrafish larvae was performed following the method described by Kim et al. (2015), with slight modifications. At 4 dpf, AB strain larvae were anesthetized with MS222 (T8910; Solarbio, China) and fixed in 4% PFA at 4 °C for 12 h. Samples were washed three times with PBS for 10 min each, dehydrated in 60% isopropanol for 30 min, and stained with 0.3% Oil Red O (G1260; Solarbio, China) working solution for 2 h at room temperature in the dark. After staining, larvae were decolorized in 60% isopropanol for 2 min and washed three times with PBS. Stained samples were imaged using a stereomicroscope.

### Whole-mount in situ hybridization of embryos

DNA fragments of *gtf3aa*, fatty acid binding protein 10a (*fabp10a*), vitamin D binding protein (*gc*), transferrin-a (*tfa*), urate oxidase (*uox*)*,* and fatty acid binding protein 2 (*fabp2*) were amplified from WT zebrafish embryos cDNAs (primer sequences were listed in Supplementary Table S4). DIG-labeled RNA probes were synthesized and purified as described in Song et al. (2020). Whole-mount in situ hybridization was carried out following established protocols (Song et al. 2021; Thisse and Thisse 2008). *gtf3aa* expression was analyzed in WT embryos at 1, 2, 3, and 4 dpf. Expression of *fabp10a*, *tfa*, and *fabp2* was examined in WT and mutant embryos at 3 dpf, while *fabp10a*, *tfa*, *gc*, and *uox* were assessed at 4 dpf.

### Western blot analysis

Total protein was extracted from 3-dpf larvae using a protein extraction kit (C510003; Sangon Biotech, Shanghai, China). Protein concentration was measured using a BCA Protein Assay Kit (CW0014S; CoWin Biosciences, Jiangsu, China). Equal amounts of protein were subjected to SDS-PAGE and Western blot analysis. Chemiluminescence detection was performed using an Immobilon Western Chemiluminescent HRP Substrate Kit (WBKLS0500; Merck Millipore, Molsheim, Germany). Primary antibodies used included Anti-RPS6 antibody (HA600084; Huabio, Hangzhou, China), Anti-RPL35 antibody (ER64837; Huabio), and Anti-β-Actin antibody (AC026; ABClonal, Hubei, China). Secondary antibodies were HRP-conjugated goat anti-rabbit IgG (AMJ-AB2003; AmyJet Scientific, Hubei, China) and HRP-conjugated horse anti-mouse IgG (7076S; Cell Signaling Technology, Boston, MA, USA).

### Polysome isolation and component analysis

At 3 dpf, *gtf3aa*^+*/*+^ and *gtf3aa*^*−/−*^ larvae (100 per group) were collected for polysome isolation as described by Choudhuri et al. (2013). Polysomes were separated using an ISCO gradient fractionator (Teledyne, El Segundo, CA, USA). Each 500 μL fraction was collected and analyzed for absorbance at 254 nm.

### RNA sequencing and transcriptome data analysis

Total RNA was extracted from 3-dpf larvae using TRIzol Reagent. RNA quantity and quality were assessed using a NanoDrop One (840–317400; Thermo Scientific, Waltham, MA, USA), Qubit fluorometer (Q33238; Invitrogen), and Agilent 2100 Bioanalyzer (Agilent Technologies, Santa Clara, CA, USA). High-quality RNA was used to construct RNA-seq libraries using the VAHTS Universal V10 RNA-seq Library Prep Kit (NR606; Vazyme), following the manufacturer’s protocol. Library quality was evaluated using the Agilent DNA 1000 kit, and sequencing was performed on a NovaSeq 6000 platform (Illumina, San Diego, CA, USA). Raw reads were quality-checked using FastQC (v0.12.0) (https://www.bioinformatics.babraham.ac.uk/projects/fastqc/) and trimmed with Trimmomatic (v0.36) (Bolger et al. 2014). Clean reads were aligned to the zebrafish reference genome (GRCz11) using the STAR aligner (v2.7.11b) (Dobin et al. 2013) in two-pass mode. Transcript reconstruction and quantification were constructed with RSEM (v2.2.1) (Li and Dewey 2011). Differential expression analysis was performed using DESeq2 (v1.34.0) (Love et al. 2014), with normalization of read counts and comparison between mutant and WT groups. Expression values were calculated as FPKM. Functional enrichment analysis of differentially expressed genes was carried out using ClusterProfiler (v4.9.0.2) (Wu et al. 2021).

### Chromatin immunoprecipitation sequencing (ChIP-seq)

mRNA encoding 3 × Flag-gtf3aa-pA was injected into one-cell stage embryos; control embryos were injected with vehicle. Embryos were collected at 36 hpf and fixed with 3.7% formaldehyde after dechorionation. ChIP-seq DNA samples were prepared using the ChIP-IT High Sensitivity kit (Cat # 53040, Active Motif, Shanghai, China), following the manufacturer’s guidelines. Primary antibodies used included Flag antibody (F1804; Sigma-Aldrich) and mouse IgG (B900620; Proteintech), each at 5 μg per sample. Input and IP samples were submitted to the Analysis and Testing Center at the Institute of Hydrobiology, Chinese Academy of Sciences (Wuhan, China), for library construction and sequencing on the NovaSeq X Plus platform. Raw reads were quality-filtered using FastQC (v0.12.0) and Trimmomatic (v0.36) (Bolger et al. 2014). Clean reads were aligned to the zebrafish reference genome (GRCz11) using Bowtie2 (v2.5.4) (Langmead et al. 2009). Read coverage was normalized using the CPM method in deepTools (v3.5.6) (Ramírez et al. 2016), and peak calling was performed with MACS2 (Zhang et al. 2008). Enriched regions were visualized and analyzed using IGV (v21.0.5) (Thorvaldsdóttir et al. 2013).

### Statistical analysis

All data analysis and figure generation were performed using R packages. Results are presented as mean ± standard deviation (SD). Statistical significance was determined using one-tailed Student′s *t* tests. Significance levels were defined as *P < 0.05 and **P < 0.01.

## Results

### Phylogenetic analysis of zebrafish Gtf3aa protein

**Fig. 1:**
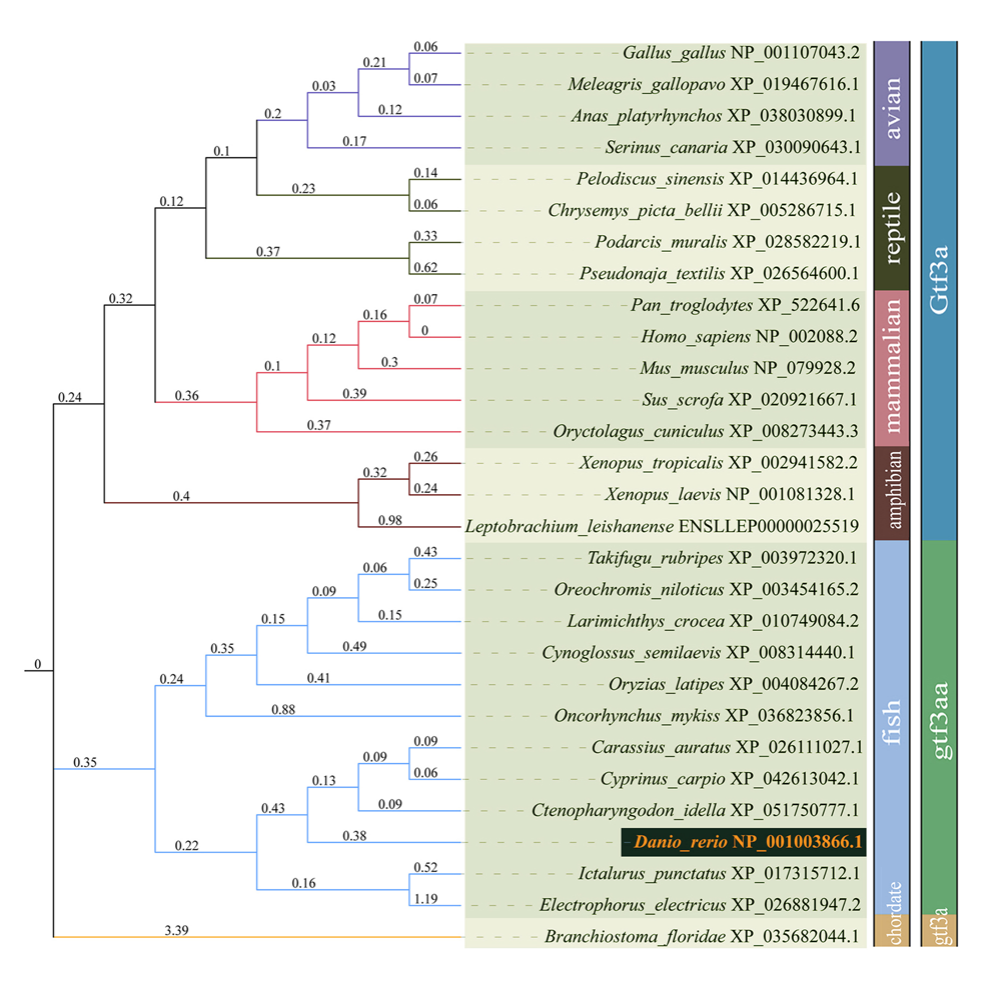
Phylogenetic analysis of Gtf3a amino acid sequences. The bar on the right indicates Gtf3a homologs, with each color corresponding to a specific protein as labeled. The penultimate bar represents classification, with each colored segment denoting a particular species as indicated. Values along the branches represent bootstrap support values

The zebrafish *gtf3aa* gene is located on chromosome 5 and comprises nine exons and eight introns. It produces two transcript variants, measuring 1575 bp (ENSDART00000049331.7) and 792 bp (ENSDART00000131665.2), respectively. The longer transcript encodes a 367 amino acid protein, whereas the shorter variant undergoes nonsense-mediated decay. Phylogenetic analysis revealed that the zebrafish Gtf3aa protein is closely related to Gtf3a proteins in amphibians and reptiles (Fig. 1). Comparative protein sequence analysis showed that zebrafish Gtf3aa shares more than 59% similarity with Gtf3a homologs in *Xenopus laevis*, *Mus musculus*, and *Homo sapiens* (Supplementary Fig. S1).

### Expression pattern of gtf3aa in early embryos and tissues

**Fig. 2:**
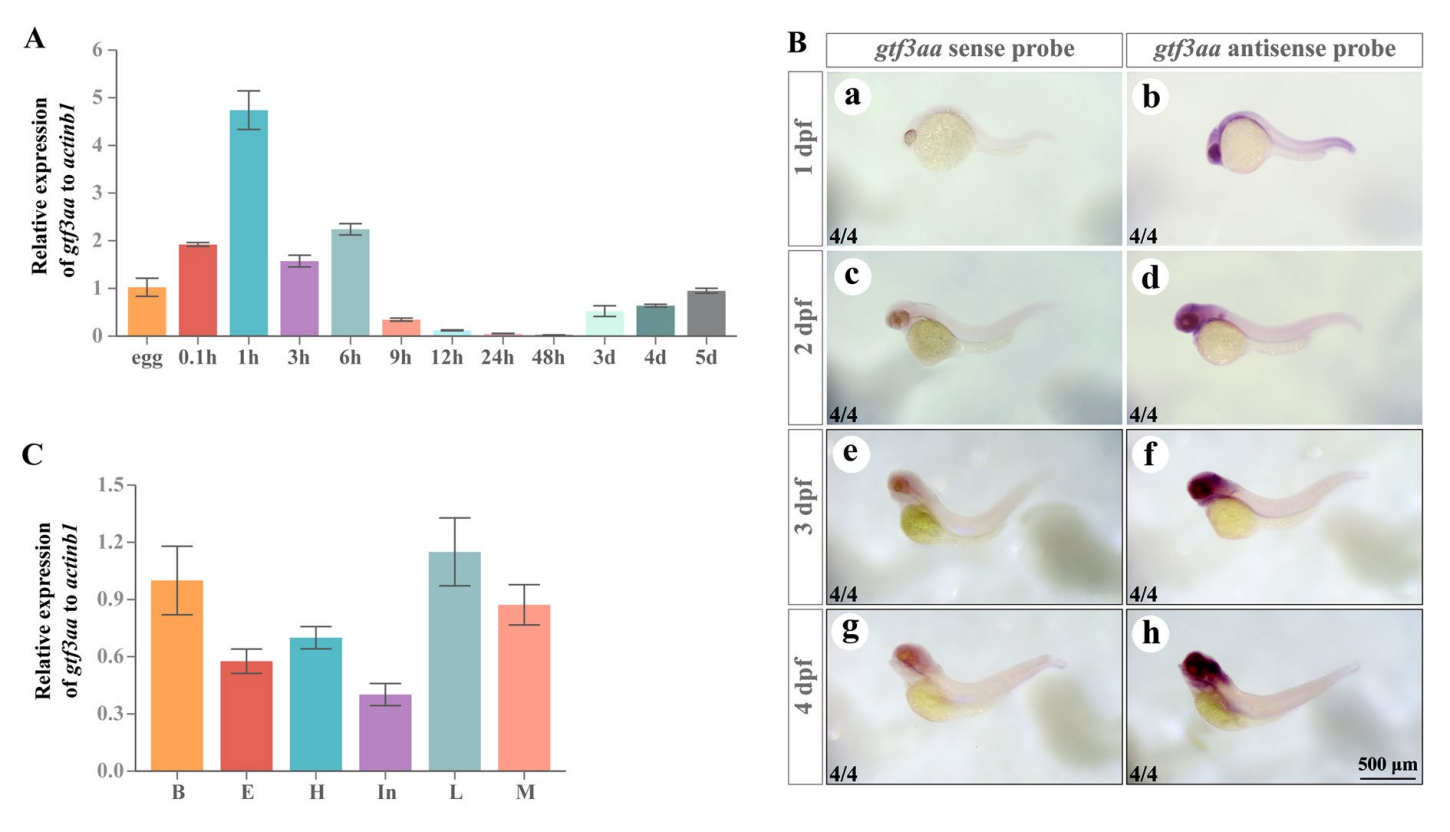
Expression patterns of *gtf3aa*. **A** Expression levels of *gtf3aa* mRNA at different stages of zebrafish embryonic development. *h* hours postfertilization, *d* days postfertilization. **B** In situ hybridization showing the localization of *gtf3aa* mRNA in 1–5 dpf zebrafish larvae. **C** Expression patterns of *gtf3aa* in various tissues of 5-dpf zebrafish larvae. *B* brain, *E* eyes, *H* heart, *In* intestine, *L* liver, *M* muscle

The expression of *gtf3aa* was analyzed during early embryonic development. The highest expression was detected at the 4-cell stage (1 hpf), followed by a gradual decline as development progressed. Between 12 and 48 hpf, *gtf3aa* expression was nearly undetectable. At 3 dpf, expression levels increased again in the larvae (Fig. 2A). In situ hybridization demonstrated that *gtf3aa* mRNA is broadly distributed across various tissues during embryogenesis, with the highest levels in the brain (Fig. 2B). Dissection and qPCR analysis of tissues from 5 dpf larvae confirmed that *gtf3aa* mRNA is highly expressed in the brain, heart, liver, and muscle (Fig. 2C).

### Gtf3aa regulates transcription of somatic 5S rRNA

**Fig. 3:**
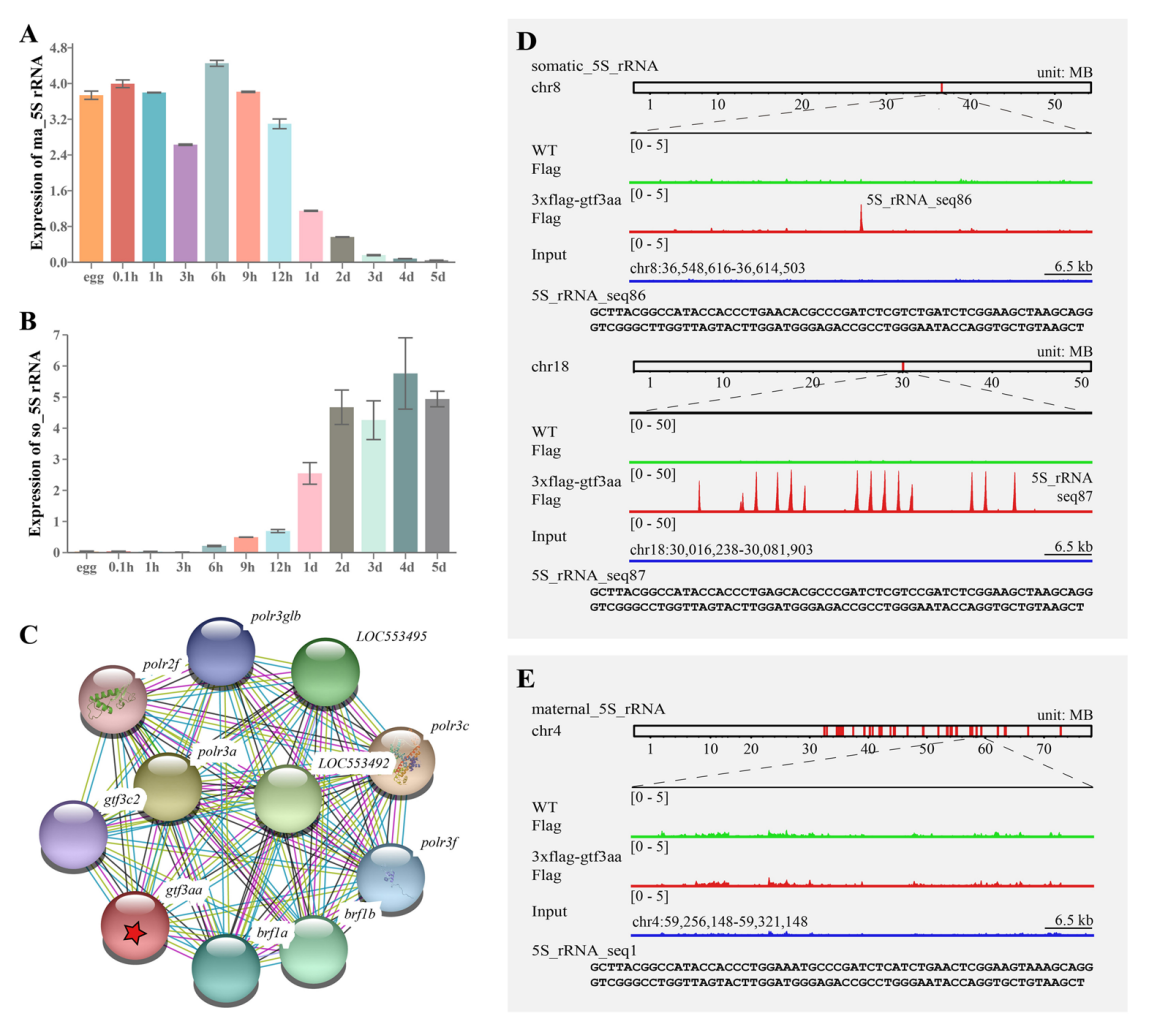
Gtf3aa binds to somatic 5S rRNA during zebrafish embryonic development. Expression patterns of maternal (**A**) and somatic (**B**) 5S rRNA during various stages of wild-type zebrafish embryogenesis. **C** Protein interaction networks for Gtf3aa. Each node represents an individual protein; colored nodes indicate the first shell of interactors. Unfilled nodes correspond to proteins with unknown three-dimensional (3D) structures, whereas filled nodes represent those with known or predicted structures. **D** IGV track view showing Gtf3aa binding at the somatic 5S rRNA gene region in 36 hpf wild-type embryos. Increased ChIP-seq peaks are highlighted with red dashed rectangles. **E** IGV track view showing Gtf3aa occupancy at the maternal 5S rRNA gene region in 36 hpf wild-type embryos

Maternal 5S rRNA is abundant in mature eggs and supports early zebrafish development. After 9 hpf, its levels begin to decline and become nearly undetectable by 3 dpf (Fig. 3A). In contrast, somatic 5S rRNA is minimally expressed in mature eggs and embryos before 6 hpf, but its levels increase beginning at 6 hpf (Fig. 3B). STRING-based protein interaction network analysis (Szklarczyk et al. 2019) showed that Gtf3aa interacts with RNA polymerase III components such as Brf1b, Polr3a, and Gtf3c2 (Fig. 3C), suggesting its involvement in transcriptional regulation. In this study, one-cell-stage zebrafish embryos were injected with 3 × Flag-gtf3aa mRNA, and ChIP-seq analysis at 36 hpf identified DNA targets bound by Gtf3aa. The results revealed specific binding to somatic 5S rDNA sequences on chromosome 8 (one copy) and chromosome 18 (14 copies) (Fig. 3D), with minimal binding to maternal 5S rDNA during development (Fig. 3E).

### Mutation of gtf3aa disrupts ribosome assembly in zebrafish

**Fig. 4:**
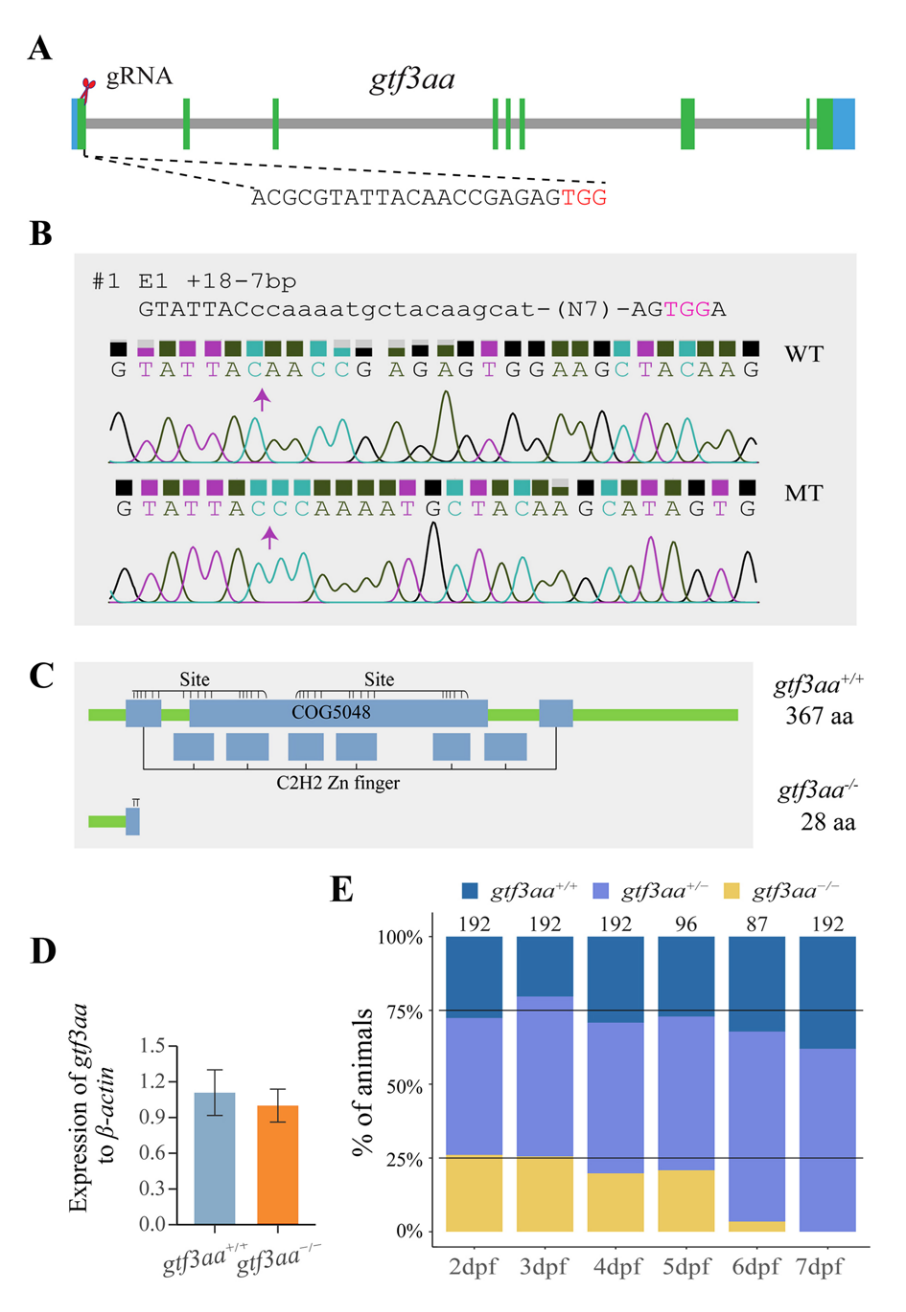
Generation and characterization of *gtf3aa* mutants in zebrafish. **A** Target site of *gtf3aa* using CRISPR/Cas9. **B** Upper panel: Sequencing chromatogram of the *gtf3aa* gRNA target site in wild-type (WT) zebrafish. Lower panel: Chromatogram of the mutant site in the *gtf3aa* genome. Insertions are shown in lowercase, and missing bases are denoted by “N” followed by the number of deleted bases in parentheses. **C** Predicted Gtf3aa protein structures from both WT and MT zebrafish. **D** Expression levels of *gtf3aa* mRNA in mutant and wild-type larvae at 3 dpf. **E** Survival rate of *gtf3aa*^+/−^ self-cross larvae. The number of larvae used at each stage is shown at the top of each bar. *WT* wild type, *MT* mutant type, *aa* amino acids, *dpf* days postfertilization

To investigate the *gtf3aa* function, a zebrafish knockout model was generated. A 7 bp deletion and an 18 bp insertion in the first exon caused a frameshift mutation, leading to premature translation termination and loss of functional protein in *gtf3aa*^*−/−*^ mutants (Fig. 4A–C). While *gtf3aa* mRNA levels in knockout larvae were reduced, the difference from WT was not statistically significant (Fig. 4D). Survival analysis of offspring from *gtf3aa*^+/−^ crosses showed a distribution (1:2:1) at 2 and 3 dpf, but the proportion of *gtf3aa*^*−/−*^ declined from 4 dpf onward, with no surviving mutants detected at 7 dpf (Fig. 4E).

**Fig. 5:**
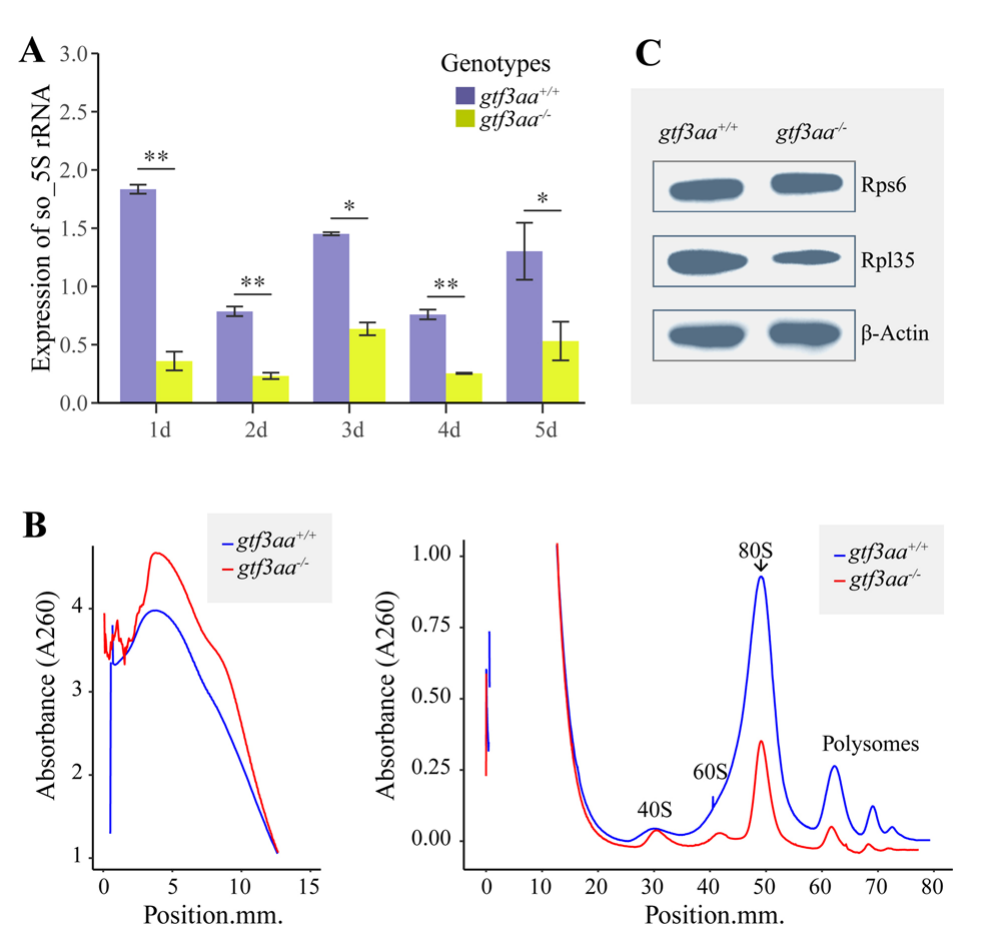
*gtf3aa* mutations disrupt 5S rRNA transcription and ribosome biogenesis. **A** Expression patterns of somatic 5S rRNA in *gtf3aa*^*−/−*^ and *gtf3aa*^+*/*+^ larvae from 1–5 dpf. 18S rRNA served as the reference gene in qPCR. **B** Polysome profiling of *gtf3aa*^*−/−*^ and *gtf3aa*^+*/*+^ larvae at 3 dpf. **C** Western blot analysis of Rps6 and Rpl35 protein levels in *gtf3aa*^*−/−*^ and *gtf3aa*^+*/*+^ larvae at 3 dpf, using β-Actin as a loading control. *so_5S rRNA* somatic 5S rRNA

Somatic 5S rRNA levels were compared between *gtf3aa*^+*/*+^ and *gtf3aa*^*−/−*^ larvae during early embryonic development (1–5 dpf). In *gtf3aa*^−/−^ larvae, somatic 5S rRNA expression did not increase significantly and remained significantly lower than that observed in *gtf3aa*^+*/*+^ larvae (Fig. 5A). Subsequently, polysome profiling was conducted to assess the levels of free ribosomes, polysomes, and ribosomal subunits in both *gtf3aa*^*−/−*^ and *gtf3aa*^+*/*+^ larvae. The analysis showed that free ribosome content was significantly elevated in *gtf3aa*^*−/−*^ mutants compared to the WT, whereas no notable difference was detected in the levels of the 40S small subunit between the two groups. In contrast, the levels of the 60S large subunit, mature ribosomes, and polysomes were significantly reduced in *gtf3aa*^*−/−*^ mutants (Fig. 5B). Western blot analysis revealed that *gtf3aa* deficiency had no substantial effect on the expression of Rps6, a component of the 40S small subunit, in zebrafish. However, the expression of Rpl35, a 60S large subunit protein, was significantly inhibited in *gtf3aa*^*−/−*^ mutants relative to WT (Fig. 5C). These findings suggest that the *gtf3aa* mutation impairs somatic 5S rRNA transcription and disrupts the assembly of the 60S large subunit, thereby causing a significant reduction in mature ribosomes and polysomes in zebrafish.

### Phenotypic characteristics of gtf3aa knockout

**Fig. 6:**
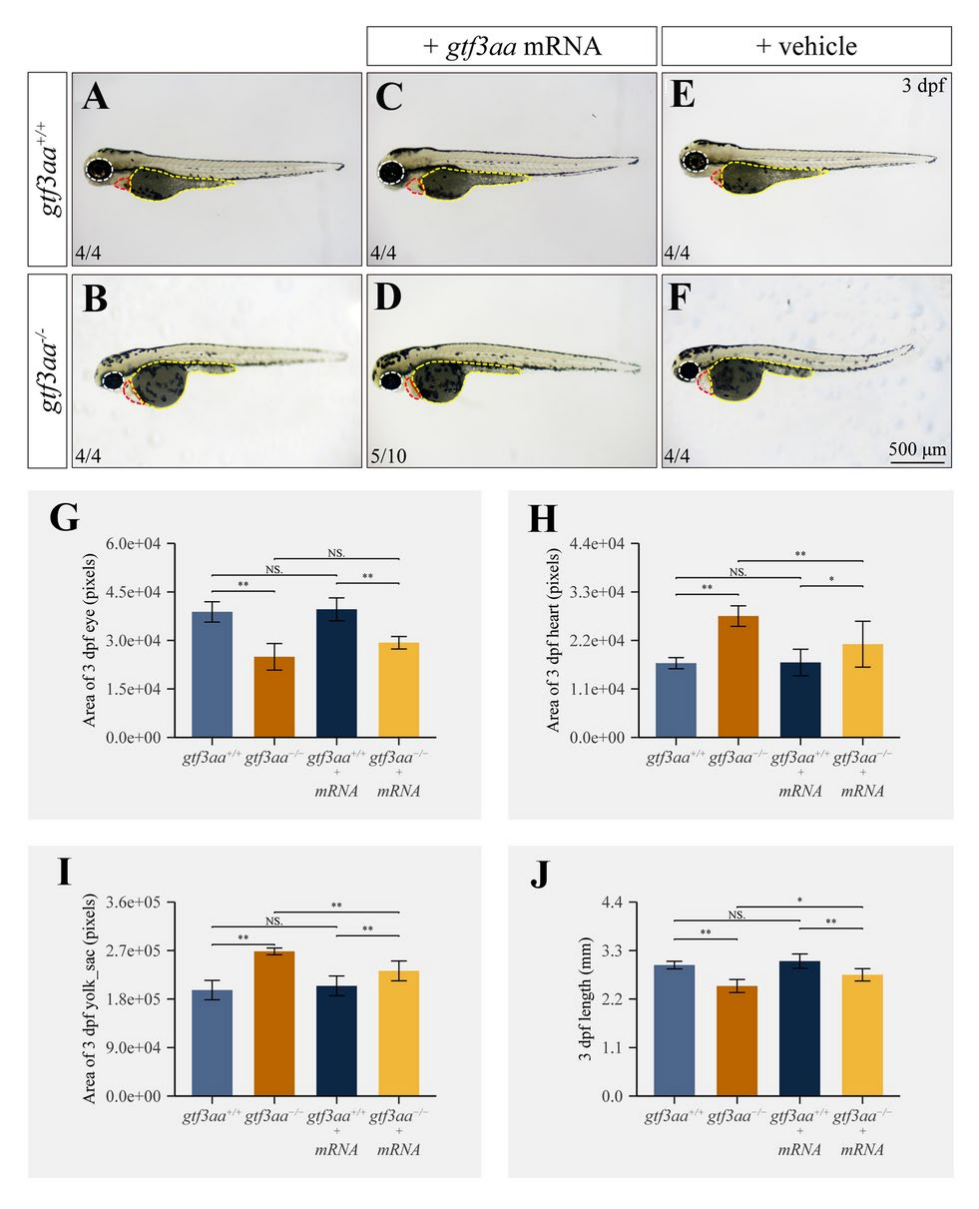
Disruption of *gtf3aa* impairs zebrafish larval development. **A**, **B** Phenotypic comparison of 3-dpf larvae between *gtf3aa*^+*/*+^ and *gtf3aa*^*−/−*^. **C**, **D** Phenotypic comparison of 3-dpf *gtf3aa*^+*/*+^ and *gtf3aa*^*−/−*^ larvae rescued by *gtf3aa* mRNA injection at the one-cell stage. **E**, **F** Phenotypic comparison of 3-dpf *gtf3aa*^+*/*+^ and *gtf3aa*^*−/−*^ larvae injected with vehicle. Eyes are outlined with white dashed lines, hearts with red dashed lines, and yolk sacs with yellow dashed lines. **G**–**J** Quantitative analysis of eye area, heart area, yolk sac area, and body length in 3-dpf *gtf3aa*^+*/*+^ and *gtf3aa*^*−/−*^ larvae

**Fig. 7:**
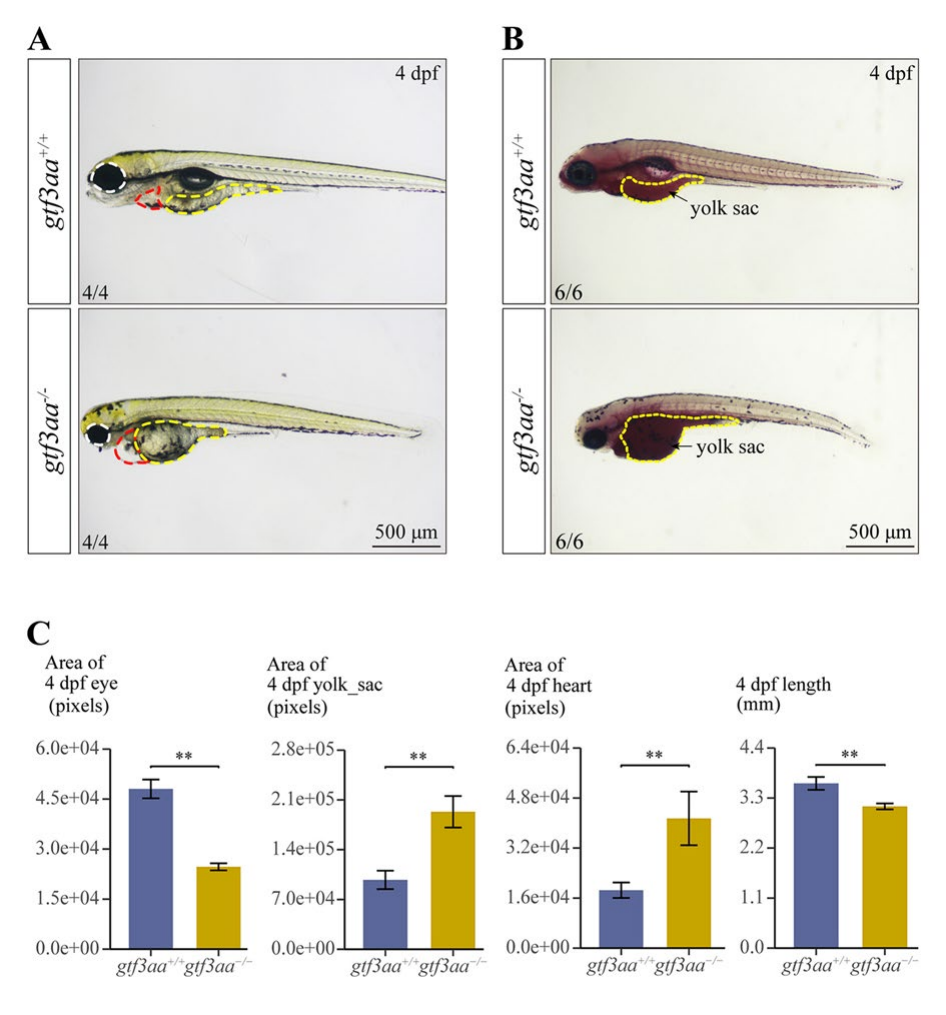
Phenotypic features of *gtf3aa* mutants at 4 dpf. **A** Morphological comparison of 4-dpf *gtf3aa*^+*/*+^ and *gtf3aa*^*−/−*^ larvae. **B** Oil red staining in 4-dpf *gtf3aa*^+*/*+^ and *gtf3aa*^*−/−*^ larvae. Yolk sacs are outlined with yellow dashed lines. **C** Quantitative analysis of eye area, yolk sac area, heart area, and body length in 4-dpf *gtf3aa*^+*/*+^ and *gtf3aa*^*−/−*^ mutants

The morphological features of tissues and organs were examined in *gtf3aa*^*−/−*^ mutants. At 3 dpf, *gtf3aa*^*−/−*^ larvae exhibited smaller eyes, enlarged pericardial cavities, larger yolk sacs, and shorter body lengths compared to *gtf3aa*^+*/*+^ larvae (Fig. 6A, B, G–J). These developmental defects were markedly alleviated following the injection of *gtf3aa* mRNA into mutant embryos, whereas the vehicle-injected control showed no significant impact on the phenotype (Fig. 6C–J). By 4 dpf, *gtf3aa*^+*/*+^ larvae had nearly completed yolk absorption, formed swim bladders, and initiated horizontal swimming. In contrast, *gtf3aa*^*−/−*^ mutants at 4 dpf displayed more severe phenotypes, including reduced eye size and enlarged pericardial cavities. Yolk absorption failed in the mutants, which also lacked swim bladder development and were unable to swim horizontally (Fig. 7A). A notable reduction in body length and eye size, along with increased pericardial cavity and yolk sac size, was clearly observed in comparison to WT counterparts (Fig. 7C). Furthermore, Oil Red O staining revealed that at 4 dpf, yolk lipid content in *gtf3aa*^+*/*+^ larvae had been almost entirely absorbed, whereas a substantial accumulation of yolk lipids persisted in *gtf3aa*^*−/−*^ mutants, indicating an inability to absorb nor metabolize these lipids (Fig. 7B). These findings suggest that loss of *gtf3aa* function lead to abnormalities in digestion, lipid absorption, and metabolic processes in zebrafish.

### Loss of gtf3aa affects the development of the liver and intestine

**Fig. 8:**
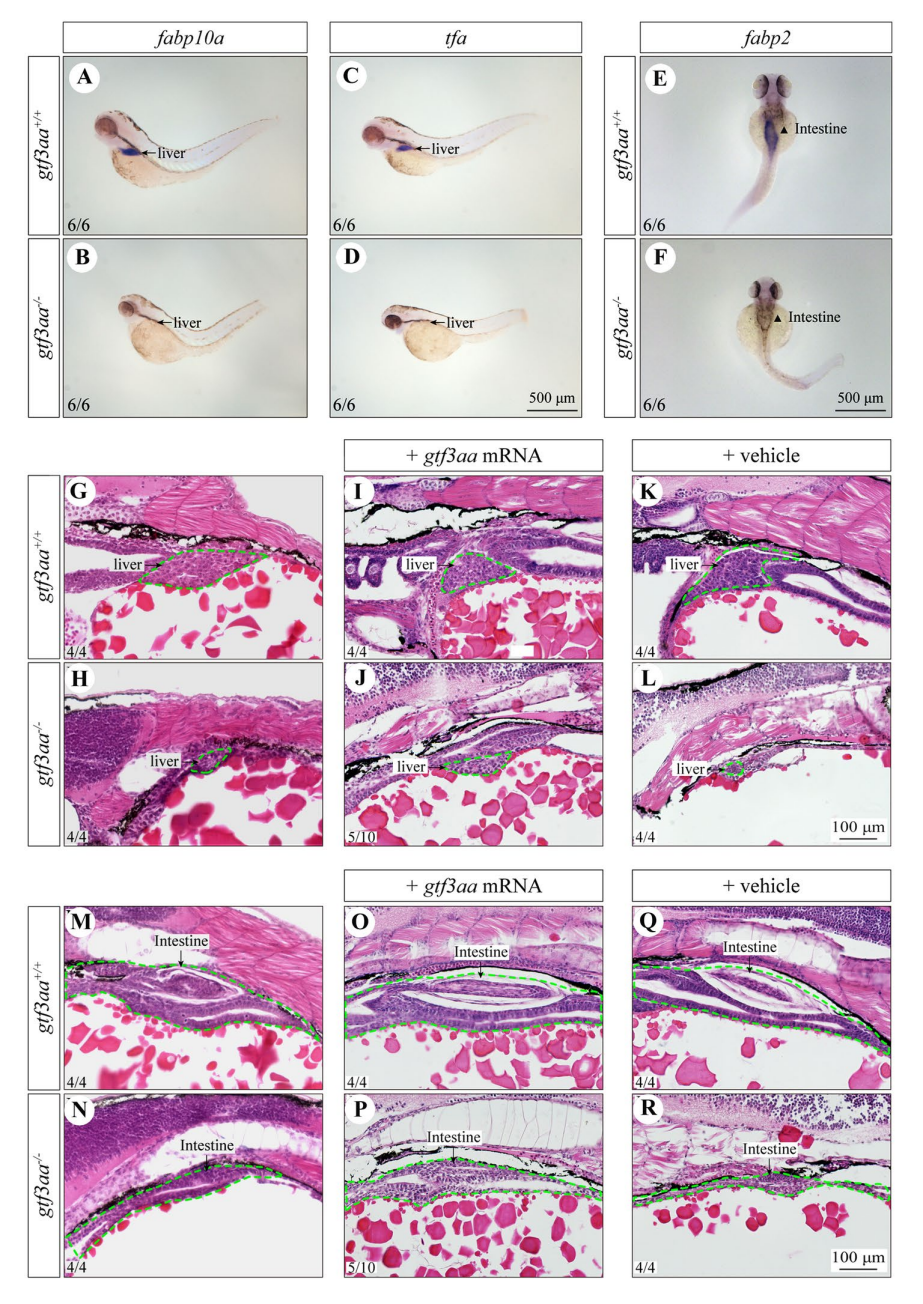
Loss of *gtf3aa* affects liver and intestine development. **A**, **B** In situ hybridization of *fabp10a* in the liver of 3-dpf *gtf3aa*^+*/*+^ and *gtf3aa*^*−/−*^ larvae. **C**, **D** In situ hybridization of *tfa* in the liver. **E**, **F** In situ hybridization analysis of *fabp2* in the intestine. **G**, **H** H&E staining of the liver in *gtf3aa*^+*/*+^ and *gtf3aa*^*−/−*^ larvae. **I**, **J** H&E staining of the liver in *gtf3aa*^+*/*+^ larvae and *gtf3aa*^*−/−*^ mutants rescued by *gtf3aa* mRNA injection at the one-cell stage. **K**, **L** H&E staining of the liver in vehicle-injected *gtf3aa*^+*/*+^ and *gtf3aa*^*−/−*^ larvae. **M**, **N** H&E staining of the intestine in *gtf3aa*^+*/*+^ and *gtf3aa*^*−/−*^ larvae. **O**, **P** H&E staining of the intestine in larvae rescued by mRNA injection. **Q**, **R** H&E staining of the intestine in vehicle-injected larvae. All comparisons are between *gtf3aa*^+*/*+^ and *gtf3aa*^*−/−*^ groups

In situ hybridization was employed to evaluate the development of major organs involved in nutrient metabolism. The liver differentiation markers *fabp10a* and *tfa* displayed strong signals in *gtf3aa*^+*/*+^ larvae but were barely detectable in *gtf3aa*^*−/−*^ mutants (Fig. 8A–D). Similarly, the intestinal marker gene *fabp2* showed robust expression in *gtf3aa*^+*/*+^ larvae but was nearly absent in *gtf3aa*^*−/−*^ larvae (Fig. 8E, F). These findings suggest that mutations in *gtf3aa* impair both liver and intestinal development in zebrafish. The morphological features of mutant tissues were further analyzed through histological sectioning. At 3–4 dpf, the livers of *gtf3aa*^+*/*+^ larvae were fully formed, whereas the livers in *gtf3aa*^*−/−*^ mutants remained at the primordium stage (Fig. 8G, H; Supplementary Fig. S2). Injection of *gtf3aa* mRNA into mutant embryos promoted liver development, partially rescuing the phenotype caused by the gene defect (Fig. 8I, J). In contrast, vehicle injection alone failed to correct the hepatic developmental abnormalities induced by the *gtf3aa* mutation (Fig. 8K, L). Additionally, *gtf3aa*^+*/*+^ larvae developed an intestinal ball at the oral end, whereas *gtf3aa*^*−/−*^ larvae exhibited a narrow intestinal tube (Fig. 8M, N; Supplementary Fig. S2). Mutant larvae injected with *gtf3aa* mRNA showed significantly larger intestinal tissue compared to untreated mutants, indicating that *gtf3aa* mRNA injection can partially rescue intestinal development defects (Fig. 8O, P). However, vehicle injection was ineffective in ameliorating the intestinal abnormalities resulting from the *gtf3aa* mutation (Fig. 8Q, R).

### Impact of gtf3aa gene mutation on gene expression in zebrafish

**Fig. 9:**
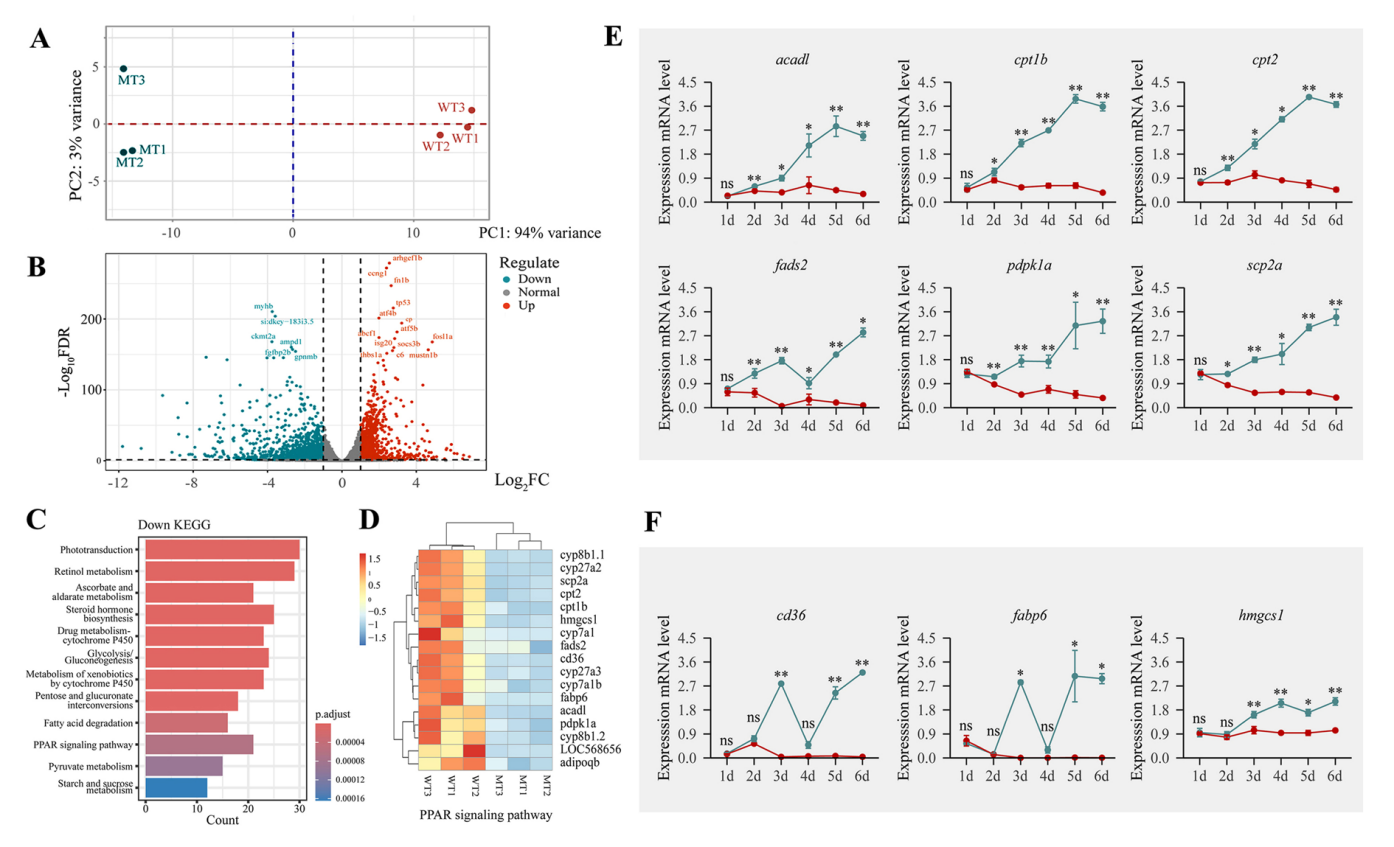
Mutations in *gtf3aa* disrupt metabolic pathways. **A** PCA of transcriptomic data from three *gtf3aa*^*−/−*^ mutants and three *gtf3aa*^+*/*+^ larvae at 3 dpf. **B** Volcano plot of differential gene expression between *gtf3aa*^*−/−*^ and *gtf3aa*^+*/*+^ larvae. **C** KEGG pathway enrichment showing downregulated pathways in *gtf3aa*^*−/−*^ mutants. **D** Heatmap of significantly downregulated genes in the PPAR signaling pathway in *gtf3aa*^*−/−*^ larvae. **E**, **F** Temporal expression of key metabolic genes in the PPAR pathway (*acadl*, *cpt1b*, *cpt2*, *fads2*, *pdpk1a*, *scp2a*, *cd36*, *fabp6*, and *hmgcs1*) from 1 to 6 dpf in *gtf3aa*^*−/−*^ compared to *gtf3aa*^+*/*+^ larvae. *β-actin* was used as the reference gene

**Fig. 10:**
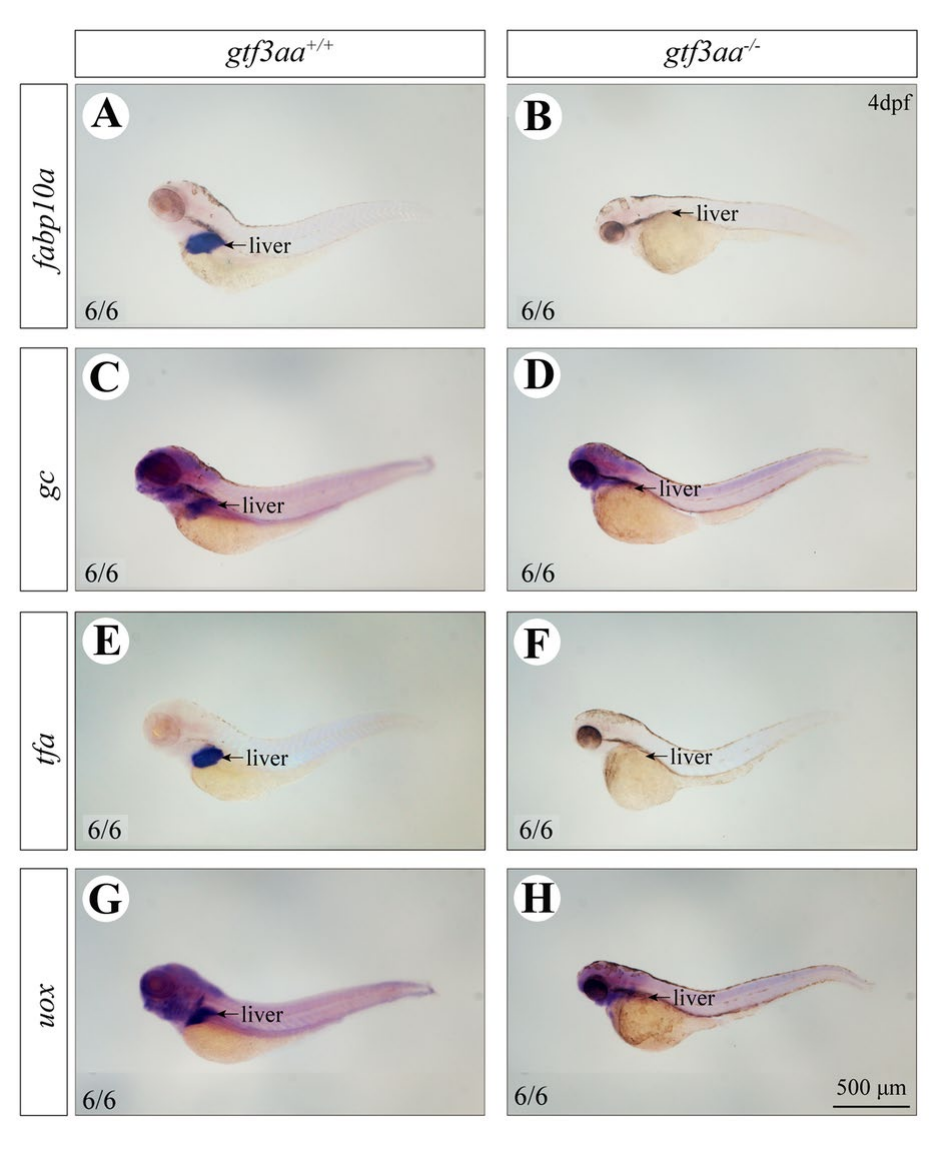
*gtf3aa* mutations impair liver metabolic functions. In situ hybridization for liver metabolic genes: *fabp10a* (**A**, **B**), *gc* (**C**, **D**), *tfa* (**E**, **F**), and *uox* (**G**, **H**) in 4 dpf *gtf3aa*^+*/*+^ and *gtf3aa*^*−/−*^ larvae. Strong signals were observed in *gtf3aa*^+*/*+^ larvae, while no signal was detected in *gtf3aa*^*−/−*^ mutants

RNA-seq analysis was conducted on *gtf3aa*^+*/*+^ and *gtf3aa*^*−/−*^ larvae at 3 dpf. Principal component analysis (PCA) showed that replicate samples clustered closely within the same group, and the two groups were clearly separated (Fig. 9A). In total, 1224 genes were significantly upregulated (log2FC ≥ 1, padj < 0.05), while 1625 genes were significantly downregulated (log2FC ≤  − 1, padj < 0.05) in *gtf3aa*^*−/−*^ larvae (Fig. 9B; Supplementary Table S5). To validate these results, four upregulated genes (*hbbe3*, *esf1*, *rsl1d1*, *thbs1a*), seven downregulated (*gnb3b*, *gngt2b*, *myhb*, *opn1sw1*, *pde6c*, *rcvrn3*, *rs1a*), and two unchanged genes (*esd* and *tubala*) were randomly selected for qPCR. The expression patterns observed by qPCR were consistent with the RNA-seq analysis (Supplementary Fig. S3). Kyoto Encyclopedia of Genes and Genomes analysis of the 1625 downregulated genes revealed that 13 of the top 15 enriched pathways were related to metabolism. These included steroid hormone biosynthesis, glycolysis/gluconeogenesis, the peroxisome proliferator-activated receptor (PPAR) signaling pathway, and primary bile acid biosynthesis (Fig. 9C). The PPAR signaling pathway is involved in lipid oxidation and metabolism in the liver and skeletal muscle, as well as in adipocyte differentiation and glucose uptake. Expression of 17 genes within the PPAR pathway was significantly decreased in *gtf3aa*^*−/−*^ mutants (Fig. 9D). To further explore this, qPCR was performed to assess the expression of PPAR pathway-related genes during early embryonic development (1–6 dpf) in *gtf3aa*^*−/−*^ and *gtf3aa*^+*/*+^ zebrafish. At 1 dpf, no significant differences were observed between the groups. However, at 2 dpf, expression of *acadl*, *cpt1b*, *cpt2*, *fads2*, *pdpk1a*, and *scp2a* had increased in *gtf3aa*^+*/*+^ larvae, whereas expression remained largely unchanged in *gtf3aa*^*−/−*^ mutants and was significantly lower in WT larvae (Fig. 9E). The expression levels of *cd36*, *fabp6*, and *hmgcs1* began increasing in *gtf3aa*^+*/*+^ larvae from 3 dpf, but showed no significant change in *gtf3aa*^*−/−*^ mutants (Fig. 9F). In situ hybridization of liver metabolic genes at 4 dpf demonstrated strong expression of *fabp10a*, *gc*, *uox*, and *tfa* in the livers of *gtf3aa*^+*/*+^ larvae, while their expression was nearly absent in *gtf3aa*^*−/−*^ mutants (Fig. 10). These results suggest that *gtf3aa* mutation suppress the expression of genes involved in lipid metabolism signaling pathways during early zebrafish development.

## Discussion

This study demonstrated that the zebrafish *gtf3aa* gene is a critical regulator of somatic 5S rRNA transcription during embryonic development. Knockout of *gtf3aa* directly impaired yolk absorption and larval growth, underscoring its pivotal role in organogenesis, particularly in the development of the eyes, heart, swim bladder, liver, and intestinal tissues. To our knowledge, this is the first study to demonstrate the in vivo role of TFIIIA gene homologs in regulating organ development in animals.

### Gtf3aa regulates somatic 5S rRNA expression and influences ribosome biogenesis

The transcription of 5S rRNA has been shown to require TFIIIA across all studied eukaryotes. In yeast, TFIIIA’s sole essential function is the transcription of the 5S rRNA gene (Camier et al. 1995). In *Xenopus*, beyond its regulatory role in 5S rRNA expression, TFIIIA interacts with nearly 50% of 5S rRNA to form the 7S particle, thereby facilitating nuclear transport and cytoplasmic storage (Ciganda and Williams 2011). In zebrafish, two 5S rRNA variants, maternal-type and somatic-type, have been identified, primarily expressed during oogenesis or in adult tissues, respectively (Locati et al. 2017). In this study, it was observed that maternal 5S rRNA accumulates in oocytes and is gradually depleted during embryonic development. In contrast, somatic 5S rRNA expression increased concurrently throughout embryonic development. The expression of *gtf3aa* initially rose, then declined during early embryogenesis, followed by a gradual increase during larval growth. Variations in *gtf3aa* expression levels corresponded with the expression pattern of somatic 5S rRNA. In zebrafish, the maternal-type 5S rDNA comprises several thousand gene copies arranged in tandem repeats with small intergenic regions on chromosome 4, whereas the somatic-type is located on chromosome 8 (one copy) and chromosome 18 (12 copies) with long intergenic regions (Locati et al. 2017). In the present study, ChIP-seq assay revealed that Gtf3aa binds to somatic-type 5S rDNA on chromosome 8 (one copy) and chromosome 18 (14 copies), including two newly identified homologous copies. No interaction was observed with the maternal 5S rDNA on chromosome 4. Furthermore, ChIP-seq analysis indicated that Gtf3aa may also bind to promoter regions of certain protein-encoded genes (e.g., *serpine2*), though with relatively low affinity. Whether Gtf3aa regulates the expression of these genes remains unclear. In *gtf3aa* knockout zebrafish, somatic 5S rRNA levels were significantly reduced. Simultaneously, the abundances of 60S large subunits, 80S mature ribosomes, and polysomes were markedly decreased. These findings suggest that *gtf3aa* plays a key role in the regulation of somatic 5S rRNA transcription and ribosome biogenesis. Similarly, TFIIIA knockdown in U2 OS cells inhibits the transcription of nascent 5S rRNA and disrupts the formation of the large 60S ribosomal subunit, leading to a reduction in 80S ribosomes and polysomes (Donati et al. 2013). Reducing TFIIIA expression to 5% of normal levels in HEK293 cells caused a threefold decrease in 5S rRNA production, leading to reduced accumulation of large ribosomal subunit rRNA and impaired ribosome biogenesis (Naesens et al. 2022; Sloan et al. 2013). These results suggest that TFIIIA modulates 5S rRNA transcription and ribosome biogenesis in a conserved manner across vertebrates.

### Mutations in genes involved in ribosome synthesis cause organ developmental defects

Disruptions in ribosomal biogenesis generally lead to defects in organogenesis and abnormal growth (Cenik et al. 2019; Danilova et al. 2011; Zhao et al. 2014; Zhu et al. 2021). In mice, mutations in the SBDS gene, which encodes a ribosome maturation factor, result in embryonic lethality (Zhang et al. 2006). In zebrafish, *Sbds* mutations cause reduced levels of 80S ribosomes, leading to stunted growth, atrophy of the pancreas, liver, and intestines, and death by 21 dpf (Oyarbide et al. 2020). Mutations in the zebrafish *rcl1* gene impair 18S rRNA maturation and ribosome assembly, resulting in death at 15 dpf (Zhu et al. 2021). In this study, the *gtf3aa* mutation in zebrafish caused a decline in mature ribosome levels and significantly impaired the development of multiple organs during late embryogenesis. Although *gtf3aa* is expressed across various tissues, its expression is highest in the brain, liver, and muscle. In *gtf3aa* mutant zebrafish, the swim bladder failed to develop, and severe impairments were observed in the heart, liver, and intestinal development. Additional defects included impaired yolk absorption, microphthalmia, and an enlarged pericardial cavity. It is hypothesized that mutations in *gtf3aa*, resulting in defective ribosome synthesis, may contribute to the observed developmental abnormalities in zebrafish. Moreover, this study demonstrated that zebrafish Gtf3aa can bind to the promoters of protein-coding genes (e.g., *serpine2*) as shown by ChIP-seq assay. Whether Gtf3aa regulates organ development through modulation of these genes remains to be investigated.

### Mutations in genes involved in ribosome synthesis lead to metabolic abnormalities

Disruptions in ribosomal biogenesis can also lead to metabolic disturbances (Zhu et al. 2021). In mice, abnormal function of ribosomal protein S6 impairs pancreatic β-cell activity, disrupting glucose homeostasis (Ruvinsky et al. 2005). Mutations in the *rrp-8* gene in *Caenorhabditis elegans* trigger nucleolar stress, affecting the FoxA/PHA-A transcription factor and causing fat accumulation (Wu et al. 2018). In humans, *RPL9* mutations impair the glycolytic pathway, redirecting metabolism toward gluconeogenesis and downregulating nucleotide biosynthesis (Lezzerini et al. 2020). In this study, we observed that expression of genes involved in metabolic signaling pathways was suppressed in *gtf3aa* mutant zebrafish. Notably, genes within the PPAR signaling pathway, which regulates lipid metabolism in the liver and skeletal muscle, were significantly downregulated. Genes promoting lipid metabolism in the PPAR pathway, including *cd36*, *fabp6*, *hmgcs1*, *scp2a*, *fads2*, *cpt1b*, *cpt2*, *acadl*, and *pdpk1a*, showed significantly reduced expression during early embryonic and larval stages in *gtf3aa* mutants compared to WT. CD36 mediates fatty acid and cholesterol uptake via endocytosis (Hao et al. 2020; Masuda et al. 2009). FABP6 is involved in cholesterol metabolism (Fang et al. 2007). SCP-2 regulates lipid and fatty acid metabolism (Xu et al. 2023). Deletion of *cpt2* inhibits fatty acid oxidation (Blackburn et al. 2019; Lee et al. 2016). The significant downregulation of these genes in *gtf3aa* mutants suggests strong inhibition of lipid metabolism in vivo. Additionally, expression of key liver metabolic genes such as *fabp10a*, *gc*, *uox*, and *tfa* was nearly undetectable in *gtf3aa* mutants. Thus, the metabolic disorder resulting from the *gtf3aa* mutation likely contributes to defects in yolk absorption and metabolic processes.

In conclusion, this study elucidates the role of *gtf3aa* in regulating somatic 5S rRNA expression and ribosome biogenesis, thereby influencing organ formation and lipid metabolism during zebrafish embryogenesis. The *gtf3aa*^−/−^ mutant zebrafish provide the first in vivo model for investigating the role of Gtf3a homologs in vertebrates. However, research on the *gtf3aa* function remains in its early stages. Further studies are needed to explore the mechanisms by which *gtf3aa*-mediated defects in ribosome biogenesis affect embryonic development and tissue formation.

## Supplementary Information

Below is the link to the electronic supplementary material.

Supplementary file1 (RAR 3362 KB)

## Data Availability

Raw RNA-seq data reported in this paper have been deposited in the NCBI Sequence Read Archive (SRA; https://www.ncbi.nlm.nih.gov/sra; BioProjectID PRJNA196811). The raw ChIP-seq data reported in this study have been deposited in the Genome Sequence Archive (GSA: CRA024228; https://ngdc.cncb.ac.cn/gsa).
